# Minimizing Narcotic Use in Rhinoplasty: An Updated Narrative Review and Protocol

**DOI:** 10.3390/life14101272

**Published:** 2024-10-07

**Authors:** Madison Mai-Lan Cheung, Anil Shah

**Affiliations:** 1 College of Medicine at Rockford, University of Illinois Chicago, Rockford, IL 61107, USA; 2 Department of Surgery, Section of Otolaryngology, University of Chicago, Chicago, IL 60637, USA; 3 Shah Aesthetics, Chicago, IL 60654, USA

**Keywords:** analgesia, pain management, opioid usage, anesthesia, opioid-sparing, postoperative pain, rhinoplasty

## Abstract

Opioids are commonly used to reduce pain after surgery; however, there are severe side effects and complications associated with opioid use, with addiction being of particular concern. Recent practice has shifted to reduce opioid consumption in surgery, although a specific protocol for rhinoplasty is still in progress. This paper aims to expand on the protocol previously established by the senior author based on updated evidence and details. This was accomplished by first high-lighting and summarizing analgesic agents with known opioid-reducing effects in the surgical field, with a particular focus on rhinoplasty, then compiling these analgesic options into a recommended protocol based on the most effective timing of administration (preoperative, intraoperative, postoperative). The senior author’s previous article on the subject was referenced to compile a list of analgesic agents of importance. Each analgesic agent was then searched in PubMed in conjunction with “rhinoplasty” or “opioid sparing” to find relevant primary sources and systematic reviews. The preferred analgesic agents included, as follows: preoperative, 1000 mg oral acetaminophen, 200 mg of oral celecoxib twice daily for 5 days, and 1200 mg oral gabapentin; intraoperative, 0.75 μg/kg of intravenous dexmedetomidine and 1–2 mg/kg injected lidocaine with additional 2–4 mg/kg per hour or 1.5 cc total bupivacaine nerve block injected along the infraorbital area bilaterally and in the subnasal region; and postoperatively, 5 mg oral acetaminophen and 400 mg of oral celecoxib. When choosing specific analgesic agents, considerations include potential side effects, contraindications, and the drug-specific mode of administration.

## 1. Introduction

Opioids are commonly used to reduce pain after surgery; however, there are severe side effects and complications associated with opioid use, with addiction being of particular concern. In 2021, a reported 269 million people used drugs worldwide, with 23% being opioid users [1]. The highest prevalence of opioid use is in North America. In the United States alone, around 50,000 people died from opioid overdoses in 2019 [1]. This number has increased every year, with 68,000 opioid-related deaths from October 2019 to October 2020 [1]. Hydrocodone and oxycodone are the cause of 77% of drug-related adverse events and are commonly prescribed for postoperative pain [2]. Notably, the rate of opioid-related deaths tends to correlate with the rate of opioid analgesic prescription following surgery, with over-prescription of opioid analgesia being a notable concern; over 70% of the opioids prescribed are unused [2]. Risk factors for developing opioid use disorder include genetic predisposition for addiction, low socioeconomic status, male gender, or White race [1].

Enhanced recovery after surgery (ERAS) is an approach to maintaining high-quality surgical care by reducing complication rates, hospital lengths of stay, and costs [3]. The specific ERAS regimen to administer is unique to each case and depends on the patient’s characteristics and type of surgery. Preoperative components include counseling, while intraoperative aspects involve administering antibiotics and maintaining euvolemia [3]. A major component of the postoperative phase is proper analgesia for pain and nausea control [3]. Many ERAS guidelines recommend multimodal analgesia to limit narcotic use and reduce side effects associated with opioids [3]. The side effects of opioids include postoperative nausea and vomiting, constipation, urinary retention, and respiratory depression [1]. Aside from various side effects, opioids also have a risk of addiction, leading to overdose. The rate of development of new persistent opioid use is 6.6% for patients who undergo plastic and reconstructive procedures [4]. In a cross-sectional study of 3017 patients who underwent otolaryngologic procedures and were prescribed opioids, the authors observed that 62.1% of patients were prescribed hydrocodone–acetaminophen, 37.0% oxycodone–acetaminophen, and 1.0% acetaminophen–codeine [5]. In rhinoplasty, 97.1% of patients are prescribed an average of 28 opioid pills [6]. The average opioid consumption within fourteen days, post rhinoplasty, is 6.15 ± 4.85 pills [7].

Recent practice has shifted towards reducing opioid consumption in surgery, although a specific protocol for rhinoplasty is still in progress. Although osteotomies can help to relieve pain for rhinoplasty patients, many patients still report mild to moderate pain [7]. Studies in the current literature suggest that a standardized regimen of seven to eleven opioid pills will sufficiently relieve postoperative pain following nasal reconstruction [7,8]. Furthermore, standard interventions to limit opioid use post rhinoplasty include, as follows: preoperatively, patient education and screening for opioid misuse; intraoperatively, the use of local nerve blocks and long-acting analgesia; and postoperatively, multimodal analgesia for pain management with acetaminophen, NSAIDs, and opioids only as needed for rescue analgesia [9]. Of note, one surgeon was able to implement a 15-component protocol on 42 patients which led to safely avoiding opioid prescriptions in 76% of rhinoplasty patients, which indicates that a no-narcotic approach to rhinoplasty is possible with some patients; however, the sample size was relatively small and 67% of patients did not provide follow-up data on analgesic compliance [10]. The protocol notably included, as follows: 4 mg of intravenous ondansetron, 300 mg of oral gabapentin, and 1 mg of scopolamine transdermal patch preoperatively; and 0.5 mg/kg intravenous ketamine bolus, continuous propofol infusion, omission of inhaled anesthetics, 10 mg of intravenous dexamethasone, local and regional block, and 30 mg of intravenous ketorolac intraoperatively [10]. This paper aims to expand on the protocol previously established by the senior author to minimize opioid consumption after rhinoplasty, based on updated evidence and details. This will be accomplished by first highlighting and summarizing analgesic agents with known opioid-reducing effects in the surgical field, with a particular focus on rhinoplasty, then compiling these analgesic options into a recommended protocol based on the most effective timing of administration (preoperative, intraoperative, and postoperative) and notable potential side effects.

## 2. Methods

The authors searched PubMed and article citations to find both systematic reviews and primary sources such as randomized controlled trials and cohort studies. The senior author’s previous manuscript, currently unpublished, on the subject was referenced to compile a list of analgesic agents of importance. Each analgesic agent was then searched in PubMed in conjunction with “rhinoplasty” or “opioid sparing”. The inclusion criteria included searching the title and abstract for keywords, including opioid-sparing, postoperative pain, multimodal analgesia, rhinoplasty, opioid use, intraoperative, postoperative nausea and vomiting, pain reduction, and pain management. For each analgesic agent, a range of 5 to 278 publications were found using the search term results. The studies were then excluded using clinical judgement of the title and abstract contact to determine relevance to a non-narcotic approach to rhinoplasty. For example, publications that observed an analgesic in a non-surgical setting or that were limited to a pediatric or neonate patient population were excluded. In total, 144 publications were included in this narrative review.

## 3. Review

### 3.1. Multimodal Analgesia

**Figure 1:**
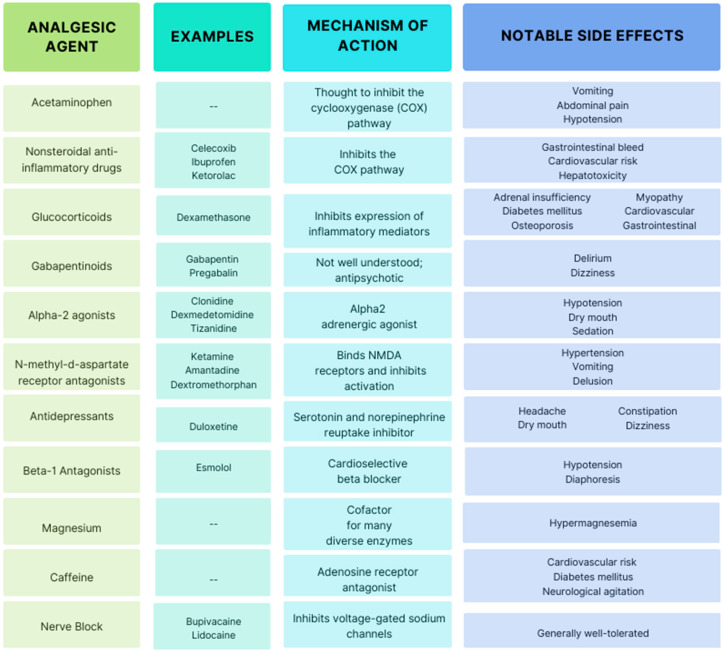
Notable non-opioid medications.

Multimodal analgesia (MMA) can be an opioid-sparing technique that utilizes a combination of analgesics with different mechanisms of action [11,12]. Alternative terms include opioid free anesthesia (OFA) or balanced anesthesia with an opioid-sparing approach [13]. Multimodal analgesia has been advocated as the pharmacologic approach of choice [11]. Considerations when implementing multimodal analgesia includes side effects, drug-drug interactions, and procedure-specific recovery pathways [14]. Potential agents with evidence of analgesic effect are included in Figure 1.

**Figure 2:**
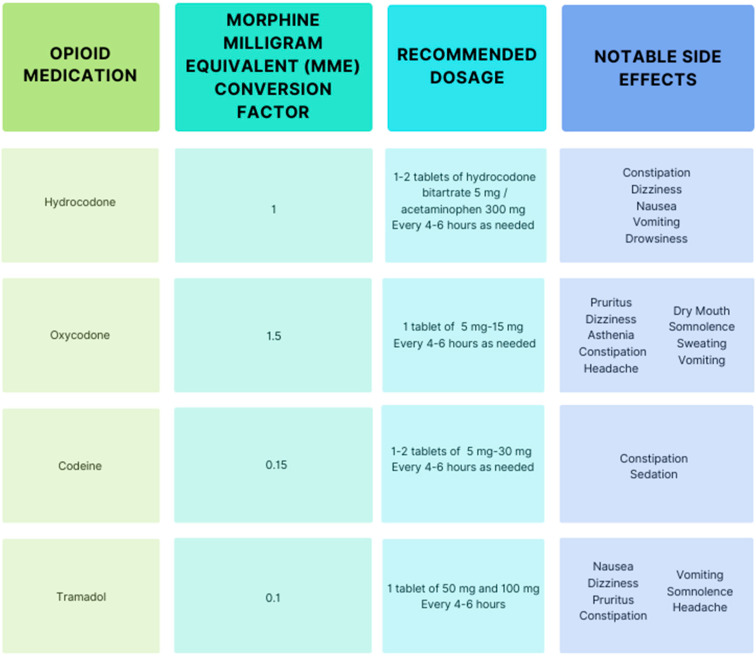
Common opioid medications [15,16,17,18,19].

In general, the most commonly prescribed oral opioids for postoperative pain management include hydrocodone–acetaminophen, oxycodone–acetaminophen, codeine, and tramadol [15]. Common opioids, their morphine milligram equivalent (MME) conversion factor, the recommended oral tablet dosage post operation, and notable side effects (excluding misuse and misuse-related effects) are included in Figure 2. Of note, the CDC recommends considering the potential side effects of increasing opioid dosage to greater than 50 MME [16].

### 3.2. Acetaminophen

Acetaminophen’s specific mechanism of action is still unclear, but its analgesic effects are thought to work by inhibiting the cyclooxygenase (COX) pathway within the central nervous system [20]. The average wholesale price of 1 g of IV acetaminophen is $56.84 while the average wholesale price of 1 g of oral acetaminophen is $10.70 [21]. The peak concentration of intravenous acetaminophen occurs 30 min faster than oral acetaminophen and results in a higher peak concentration in plasma than equivalent oral doses [22]. However, in orthopedic surgery, the higher concentration of IV acetaminophen at a faster rate has not been shown to be clinically significant [23].

IV acetaminophen has been found to have limited clinical benefit compared to oral or rectal formulations [24]. There was found to be no difference in pain relief between IV and oral acetaminophen for gynecologic surgery [25]. There has also been shown to be no significant difference in length of stay, patient satisfaction, or opioid consumption between IV and oral acetaminophen following laparoscopic surgery [26].

Oral acetaminophen is regularly prescribed for postoperative pain in rhinoplasty patients and is taken concurrently with oxycodone. In these cases, oxycodone is mostly used within the first two days post operation with a decline, while acetaminophen is used for the first five days post operation before a decline [27]. Additionally, an average of 5 tablets of 5 mg acetaminophen were used by patients following rhinoplasty [27]. Similarly, 1000 mg of preemptive oral acetaminophen has been used as a standard analgesic regimen for sufficient pain control in rhinoplasty [28].

Notably, adverse side effects within 72 h include vomiting, abdominal pain, and hypotension [29]. Acetaminophen can be hepatotoxic and has been associated with agranulocytosis, renal failure, coagulopathy, and encephalopathy [29].

### 3.3. Nonsteroidal Anti-Inflammatory Drugs (NSAIDs)

#### 3.3.1. Celecoxib

Celecoxib appears to be effective in reducing opioid consumption after rhinoplasty. The mechanism of action for celecoxib involves inhibiting the synthesis of prostaglandin and it is selective for COX-2 inhibition [30]. In one study, patients who were given celecoxib took 26.5 fewer oral morphine equivalents than the patients who did not [31]. In a study of 994 patients in noncardiac surgery, celecoxib was found to decrease nausea and vomiting without any increase in intraoperative bleeding [32]. After plastic surgery, it was determined that 400 mg of oral celecoxib on the day of surgery and three days postoperatively is effective after surgery [33]. In rhinoplasty specifically, a study demonstrated a 76.2% decrease in opioid use and an 83.4% decrease in incidences of nausea/vomiting when patients were given 200 mg of celecoxib twice daily for 5 days prior to surgery [32]. However, head-to-head, oxycodone–paracetamol was found to be more effective in improving postoperative pain than celecoxib, which indicates that, in some cases, celecoxib may be insufficient for the relief of postoperative pain [34]. As with all NSAIDs, the side effects of celecoxib include increased cardiovascular risk, including stroke and myocardial infarction, as well as potential gastrointestinal damage such as bleeding and ulceration [35,36]. Celecoxib is metabolized by CYP2C9, which indicates possible drug–drug interactions with medications that inhibit CYP2C9 such as fluconazole [37].

#### 3.3.2. Ibuprofen

Ibuprofen is an over-the-counter anti-inflammatory commonly used for mild to moderate pain [38]. Ibuprofen non-selectively inhibits COX-1 and COX-2 and thus inhibits prostaglandin synthesis [38]. In a study of 99 orthopedic trauma patients, intravenous ibuprofen was shown to improve pain, lengthen the time until narcotic use, and reduce opioid usage compared to placebo [39]. There is support for the administration of pre-emptive intravenous ibuprofen prior to septorhinoplasty; compared to placebo, pre-emptive ibuprofen had lower postoperative pain and decreased fentanyl consumption [40]. Compared to preoperative paracetamol, the administration of 800 mg of intravenous ibuprofen in 100 mL of saline solution prior to rhinoplasty more significantly reduced postoperative pain [41]. Adverse side effects include gastrointestinal bleeding, nephrotoxicity, hypersensitivity reaction (rash), small increases in systolic blood pressure, and Reye’s syndrome in children [38]. Due to the increased risk of bleeding, ibuprofen use following rhinoplasty has been limited in the past; however, a literature review reported no significant difference in bleeding events or pain between ibuprofen and other pain management in four randomized controlled trials in plastic surgery [42].

#### 3.3.3. Ketorolac

Ketorolac is often used for the management of moderate-to-severe pain in synergy with opioids to decrease total opioid use [43]. Ketorolac inhibits both COX-1 and COX-2 and is shown to have higher potency than other NSAIDs [43]. Like other NSAIDs, potential side effects include gastrointestinal bleeding, increased cardiovascular risk, nephrotoxicity, hepatotoxicity, and increased bleeding risk [44]. Ketorolac was shown to have equal effectiveness as morphine in decreasing postoperative pain; ketorolac was additionally cheaper and led to faster discharges in comparison to morphine, improving efficiency overall in the emergency department [45].

In general, NSAIDs can be harmful to the gastrointestinal system and can cause serious kidney damage and lead to bleeding when used in conjunction with glucocorticoids [46]. NSAIDs have been shown to increase blood pressure and increase the risk of acute kidney injury when taken with diuretics, ACE inhibitors, or angiotensin receptor blockers (ARBs) [43]. There have additionally been reports of interactions between NSAIDs and anti-thrombotic drugs, such as aspirin and warfarin, which can lead to an increased risk of gastrointestinal bleeding [43]. The risk of gastrointestinal bleeding, particularly hematemesis, further increases when an individual taking NSAIDs drinks any amount of alcohol [47]. Bleeding risk is additionally increased when NSAIDs are co-administered with selective serotonin reuptake inhibitors (SSRIs) due to SSRIs’ inhibiting of platelet adhesion [43]. It is also necessary to be cautious of the concomitant use of lithium with ketorolac, which leads to decreased lithium clearance and an increased risk of lithium toxicity [43].

### 3.4. Glucocorticoids

Glucocorticoids act by binding glucocorticoid receptors that translocate to the nucleus and alter gene transcription, which in turn inhibits the expression of inflammatory mediators such as interleukin-10 and interleukin-1 receptor antagonist [48]. During surgery, patients undergo a stress response with activation of the hypothalamus–pituitary axis and an increase in proinflammatory cytokines, corticotropin-releasing hormone, and adrenocorticotropic hormone, which leads to an increase in cortisol, an endogenous glucocorticoid [49]. Low-dose dexamethasone is often used perioperatively to reduce postoperative levels of these cytokines, as well as to decrease pain, fatigue, and nausea [50]. However, in patients taking chronic steroid therapy (prednisone 5–10 mg/day over three weeks), there is an increased risk of developing tertiary adrenal insufficiency; in patients taking prednisone greater than 20 mg for longer than three weeks prior to surgery, glucocorticoids are given perioperatively on a stress–dose regimen (the dose of glucocorticoid should be equivalent to the amount of cortisol estimated to be secreted in the normal physiological response) [49]. There is conflicting evidence regarding the proper guidelines and benefits of the stress–dose glucocorticoids; overall, a baseline maintenance dose of steroid therapy should be implemented [51].

Glucocorticoids are also commonly used perioperatively to reduce postoperative nausea and vomiting (PONV). In a recent systematic review and meta-analysis of 47 randomized controlled trials, perioperative administration of high-dose glucocorticoid was determined to be an effective prophylaxis for PONV and decreased opioid consumption compared to placebo [52]. However, there was no statistical difference in patient-reported pain [52]. Furthermore, when added to the recommended base treatment for post-surgical pain of paracetamol and NSAIDs, a systematic review showed glucocorticoids provided little to no difference in opioid consumption and pain, although there was uncertainty with respect to the evidence [53]. Preoperative glucocorticoids are additionally beneficial in decreasing complications and lengths of hospital stay [54]. With regards to rhinoplasty patients, methylprednisolone reduces periorbital ecchymosis and oedema compared to placebo. However, because of the immunosuppressive effect of glucocorticoids, there is evidence that chronic systemic corticosteroids can lead to an increase in surgical site infections [55]. Other adverse side effects include cushingoid features, adrenal insufficiency, osteoporosis, diabetes mellitus, myopathy, glaucoma, and increased cardiovascular, gastrointestinal, and dermatologic risk [56]. Adverse effects increase with high doses and chronic use and are seen in 90% of patients who take corticosteroids for more than 60 days [57].

### 3.5. Gabapentinoids

Gabapentinoids (gabapentin and pregabalin) are anticonvulsant medications most often used for chronic neuropathic pain. The mechanism of action is not well understood [58]. In a meta-analysis of seven randomized controlled trials involving gabapentin as an adjunct for management of post-spinal surgery pain, gabapentin was associated with decreased postoperative pain; patients had a reduced need for morphine [59]. Gabapentin was also found to decrease the use of fentanyl and was found to be safe and effective for pain following cesarean delivery [60]. Preoperative gabapentin administered 1 h prior to mastectomy led to decreased morphine use and increased time to administration of a postoperative analgesic [61]. In studies of single-dose gabapentin prior to rhinoplasty, it was found that administering a single dose of gabapentin 600 mg preoperatively decreased restlessness during recovery and extubation; however, there was not a significant decrease in postoperative pain, nausea, and dizziness [62]. There is evidence that when the dose of pre-rhinoplasty gabapentin was increased to 1200 mg, there was a reduction in postoperative analgesic use, opioid use, and an increase in time to first analgesic request [63,64]. In these cases, there was a significant report of dizziness in patients who took gabapentin versus placebo [64].

There are some considerations when using gabapentin in combination with opioids. In a cohort study of surgical admissions in the United States, the absolute risk of overdose was 1.4 per 10,000 patients with gabapentin exposure compared to 0.7 per 10,000 patients who received opioids only [65]. Gabapentin is additionally contraindicated in pregabalin use and antacids containing magnesium and aluminum hydroxides [58]. Gabapentin was also associated with a higher rate of delirium, new antipsychotic use, and pneumonia in surgical patients aged 65 years or older compared to patients who did not consume gabapentin [66]. Other side effects to be aware of include teratogenicity, hypoventilation, myopathy, and respiratory failure [67].

### 3.6. Alpha-2 Agonists

#### 3.6.1. Clonidine

Clonidine is commonly used as an antihypertensive agent for its action as an alpha-adrenergic receptor agonist [68]. Studies on the use of clonidine as a perioperative anti-analgesic provide conflicting results. In a study observing noncardiac surgical patients, the use of clonidine perioperatively did not decrease opioid use or postoperative pain [69]. However, a systematic review and meta-analysis of ten randomized clinical trials demonstrated an improvement in pain and nausea and vomiting following ophthalmic surgery in children, with little bleeding risk [70]. Clonidine has also been shown to decrease postoperative pain and morphine consumption following colorectal surgery by reducing cytokine response [71]. There are limited studies of clonidine use during rhinoplasty, although there is some evidence of decreased bleeding times during surgery when oral clonidine is administered as a pre-anesthetic [72,73].

The use of low-dose clonidine in noncardiac surgery has an increased risk of clinically important hypotension and nonfatal cardiac arrest [74]. Other common side effects of clonidine include dry mouth and sedation [75].

#### 3.6.2. Dexmedetomidine

Dexmedetomidine is a specific alpha2-adrenergic agonist with analgesic properties [76]. In general, dexmedetomidine has been shown to be comparable with propofol in maintaining sedation [77]. Evidence suggests that dexmedetomidine can have some opioid effects. The continuous administration of dexmedetomidine has been shown to lead to an increased effect of local anesthesia and decreased the use of postoperative opioids [78,79]. There have been observed decreases in pain, nausea, vomiting, and time to initial analgesia request in patients receiving dexmedetomidine with general anesthesia compared to remifentanil [80]. In rhinoplasty, dexmedetomidine and remifentanil were shown to have comparable patient satisfaction, extubation time, recovery time, and additional analgesic requirement [81]. Additionally, 0.75 μg/kg of intravenous dexmedetomidine with morphine and propofol was associated with a shorter procedure time, higher patient satisfaction, and lower pain scores, with no adverse side effects [82]. On the other hand, a systematic review of seven studies demonstrated that perioperative dexmedetomidine did not significantly lower postoperative pain compared to placebo; however, it was noted that these studies consisted of low-quality evidence due to imprecision and methodological limitations [83]. Dexmedetomidine can also be used preoperatively, which decreases postoperative opioid use [84]. Additionally, dexmedetomidine can be added to bupivacaine to extend nerve blocks and is of benefit as an adjunct to epidurals [85].

Notably, dexmedetomidine should be given over a minimum 10 min period to limit the risk of hypertension, bradycardia, and asystole [15]. Other side effects include hypotension, nausea, atrial fibrillation, and hypoxia; these symptoms are associated with the loading of dexmedetomidine [86].

#### 3.6.3. Tizanidine

Tizanidine is a centrally acting alpha2-agonist that has an additional role as a muscle relaxant [87]. Tizanidine has been shown to decrease postoperative pain and analgesic use following inguinal hernia repair [87]. Oral tizanidine has also been observed to pre-emptively reduce intraoperative opioid consumption; however, there was no significant effect on reducing postoperative opioid use [88]. Side effects include dry mouth, nausea, vomiting, and dizziness [88].

### 3.7. N-Methyl-D-Aspartate (NMDA) Receptor Antagonists

#### 3.7.1. Ketamine

Ketamine is an NMDA receptor antagonist commonly used for its potent analgesic activity while also maintaining cardiovascular and respiratory functions [89]. When given perioperatively, intravenous ketamine reduces pain 24 h after a variety of surgeries (abdominal surgery, thoracotomy, gynecologic surgery, arthroscopic anterior cruciate ligament repair, cardiac surgery, laparoscopic cholecystectomy, lumbar spinal fusion surgery, radical prostatectomy, and hemorrhoidectomy) and decreases opioid requirements [90,91]. Contrastingly, an alternative systematic review and meta-analysis demonstrated that ketamine was effective for pain reduction at 15 min, but that, overall, it did not have a statistically significant difference compared to opioids [92]. In studies administering low-dose intravenous ketamine during septorhinoplasty, ketamine was shown to be very effective at reducing emergence agitation rates, rescue opioid analgesics use, and postoperative pain compared to saline [93,94]. Thus, the administration of ketamine for managing pain after rhinoplasty needs to be further established. Side effects most commonly affect younger patients and include hypertension, vomiting, and delusion [95]. A recent systematic review and meta-analysis also revealed increased reports of hallucinations after surgery in patients administered ketamine [96].

#### 3.7.2. Amantadine

There is limited evidence supporting amantadine as an opioid-sparing analgesic. Perioperative oral amantadine reduced morphine use, postoperative pain, and plasma clearance of morphine following prostatectomy and elective spinal surgery [97]. However, in a study on preoperative oral amantadine in mandibular fracture surgery, there was no difference in nausea, vomiting, postoperative pain, or analgesic use [98]. The effect of amantadine in rhinoplasty has yet to be determined.

#### 3.7.3. Dextromethorphan

There are numerous studies in support of dextromethorphan in postoperative pain relief. In spinal surgery, perioperative oral dextromethorphan improved the total use of analgesics during surgery and reduced pain in the first four hours after surgery when added to morphine [99]. Furthermore, in a study on hysterectomy, dextromethorphan was shown to have significant effects compared to placebo on reducing pain scores at rest, morphine use in the first 24 h, and oral analgesic consumption in the following 48 h. In a systematic review and meta-analysis of 14 randomized controlled trials, dextromethorphan reduced opioid consumption from 24–48 h after surgery and postoperative pain from 1–24 h [100]. With regard to rhinoplasty, when given 90 min prior to surgery, 45 mg of oral dextromethorphan was seen to reduce pain scores during packing removal and in the post-anesthesia care unit, as well as to decrease morphine use [101]. Sixty to sixty-four percent of patients experience side effects at doses greater than 4 mg/kg [102]. Common side effects of dextromethorphan include feeling drunk, nystagmus, nausea, vomiting, dizziness, blurry vision, and ataxia [102].

### 3.8. Duloxetine

Duloxetine is a serotonin and norepinephrine reuptake inhibitor often used as an antidepressant or for chronic pain relief [103]. Duloxetine has not been well studied in the surgical setting. In a systematic review and meta-analysis of eight randomized controlled trials for total knee arthroplasty or total hip arthroplasty, there was significant evidence that duloxetine reduced perioperative opioid use and PONV [104]. There was, additionally, moderate evidence that duloxetine decreased pain three weeks postoperatively [104]. In mastectomy patients, there is evidence that perioperative duloxetine decreases total opioid consumption at 24 h but does not significantly decrease PONV compared to placebo [105]. There is some evidence that duloxetine is associated with postoperative drowsiness [104]. There is little evidence that, when duloxetine is administered with anticoagulants such as warfarin, there is an increased risk of bleeding during operation [103]. Other side effects are common to antidepressants, including headache, dry mouth, constipation, and dizziness [103].

### 3.9. Esmolol

Esmolol is a cardio-selective beta antagonist administered intravenously [106]. Notably, a systematic review of nineteen randomized controlled trials found that numeric postoperative pain scores were reduced by 1.16 out of 10, opioid rescue dosing was reduced by 69%, PONV decreased by 61%, and intraoperative opioid administration was decreased when esmolol was administered as an adjunct to general anesthesia [107]. In rhinoplasty, there is support that intraoperative esmolol decreases opioid use and pain scores three hours after surgery compared to normal saline [108]. Notable side effects of esmolol include hypotension and diaphoresis [106].

### 3.10. Magnesium Sulfate

Magnesium is a positively charged cation absorbed from a regular diet and has many diverse physiological roles, including binding voltage-gated calcium ion channels at the neuromuscular junction [109]. The use of intravenous magnesium sulfate to reduce postoperative pain has produced conflicting evidence. In one systematic review of twenty randomized clinical trials, perioperative magnesium reduced both postoperative pain and opioid use with no report of clinical toxicity [110]. However, a different systematic review reported that magnesium helped to manage postoperative pain and limited opioid use but did not necessarily decrease opioid-related side effects [111]. Magnesium sulfate is generally well-tolerated, with potential side effects at high doses including hypermagnesemia, minor flushing, and worsening of neuromuscular disease symptoms [109].

### 3.11. Caffeine

Caffeine is a central nervous system stimulant that antagonizes adenosine receptors and is legal globally [112]. Caffeine consumption has often been documented as an analgesic adjuvant. About 5–10% more patients reported postpartum or postoperative dental pain relief when taking at least 100 mg caffeine in addition to paracetamol or ibuprofen [113]. The analgesic properties of caffeine have not been well studied in the setting of surgery; however, one randomized controlled trial indicated that administering intravenous caffeine during surgical closure after laparoscopy did not reduce postoperative opioid consumption, although it was well-tolerated [114]. In general, excessive caffeine can lead to cardiovascular risk, diabetes mellitus, neurological agitation, decreased tone of the esophageal sphincter, and miscarriage risk [115].

### 3.12. Nerve Blocks

Perioperative nerve blocks have been used in a variety of procedures, particularly orthopedic surgeries, to prevent postoperative pain [116,117]. Nerve blocks limit the production of action potentials by nerves by inhibiting sodium channels along the neuronal membrane [116]. They can be administered in a single shot or through a catheter for continuous administration [118]. Compared to opioids, nerve blocks lead to reduced postoperative pain, nausea, vomiting, sedation, and pruritus [119]. Further literature supports the addition of dexmedetomidine and dexamethasone to prolong the effects of nerve blocks. Compared to local anesthetic alone, dexmedetomidine in addition with local anesthetic increased the postoperative duration of nerve block for five hours [120]. Dexamethasone increased sensory and motor nerve block effects by 144 min and 180 min, respectively [121]. Nerve blocks are generally well-tolerated without major complications, although generally there is the potential risk for hematoma, infection, nerve damage, and systemic toxicity [122]. Nerve injury and local anesthetic systemic toxicity are uncommon but possible complications [116].

#### 3.12.1. Bupivacaine

Bupivacaine is commonly used in orthopedic surgery to reduce postoperative pain. Ultrasound-guided brachial plexus blocks with 0.25% bupivacaine have been shown to produce analgesic effects that last up to 21.95 h without complications [123]. In hand surgery, lysosomal bupivacaine was shown to reduce pain for 2.2 days post operation and decrease opioid prescription rates (20.4% compared to 59–76% in patients without nerve block) [124]. Side effects were rare (6.8%) and were limited to nausea, lightheadedness, and vertigo [124].

In a study by the senior author on perioperative nerve blocks in the immediate postoperative period following rhinoplasty, a 1.5 cc total bupivacaine nerve block was performed in three distinct areas, with a nerve block along the infraorbital area bilaterally as well as in the subnasal region; additionally, 1.5 cc total was reapplied to the infraorbital nerves and subnasal region postoperatively [125]. Forty-two percent of patients who did not receive a nerve block needed additional opioids compared to 2.5% in the patients who did receive a nerve block [125]. Those in the nerve block group had decreased their postoperative recovery time by 74 min. Additionally, 42.5% of patients who were not administered a nerve block required antiemetics [125]. In comparison, 7.5% of those who received a nerve block required an antiemetic [125]. Overall, there was a shorter recovery time in patients who were administered a nerve block [125]. A systematic review and meta-analysis found that nerve blocks to the infraorbital nerve, sphenopalatine ganglion, external nasal nerve, central facial nerve, and total nerve were all associated with a significant decrease in postoperative pain, opioid requirements, and a longer duration until first analgesia request [122].

#### 3.12.2. Lidocaine

Lidocaine inhibits voltage-gated sodium channels and reduces inflammatory mediators; it is commonly used as a local anesthetic due to its safety and efficacy [126]. Furthermore, as a nociceptive antagonist, lidocaine has analgesic properties and is commonly used to reduce pain following invasive or surgical procedures [126]. The recommended dose is 1–2 mg/kg intravenous lidocaine initially, then continuous administration of 2–4 mg/kg per hour [126]. Lidocaine can be administered intravenously, topically, or by inhalation [127]. Generally, the addition of local infiltration anesthetic to general anesthesia preoperatively decreases analgesia use and complications post-surgery [128]. Lidocaine patches have been shown to decrease postoperative pain at 12, 24, and 48 h after surgery and reduce postoperative opioid use [129]. In the setting of rhinoplasty, intravenous lidocaine has been shown to decrease postoperative pain and emergence agitation compared to placebo [130]. Furthermore, rescue analgesia administration and postoperative nausea were significantly decreased in patients administered intraoperative intravenous lidocaine compared to saline [127]. However, there is evidence that alternative analgesic agents, including levobupivacaine or tramadol, are more effective at reducing postoperative pain scores [128,131,132]. Lidocaine is generally well-tolerated, but notable side effects include contact dermatitis and allergic reactions, as well as cardiotoxicity when administered as an intravascular injection.

#### 3.12.3. Total Intravenous Anesthesia vs. Inhalation Anesthesia

Total intravenous anesthesia (TIVA) has been compared to inhalation anesthesia to determine the most suitable method for reducing perioperative risk and patient pain. In a systematic review of three randomized controlled trials of robotic-assisted laparoscopic radical prostatectomy, there was no significant difference between postoperative pain [133]. In head and neck surgery, TIVA was shown to have fewer pulmonary complications, including pulmonary edema, pneumonia, or atelectasis [134]. On the other hand, there is evidence that inhaled anesthesia is associated with fewer surgical complications, including wound abscess, sepsis, and anastomotic leak, compared to TIVA [135]. In rhinoplasty, TIVA is associated with reduced pain recovery time and PONV, although there was no significant difference in postoperative or surgical complications between TIVA and inhaled anesthesia [136]. When compared to inhaled sevoflurane anesthesia, TIVA reduced emergence agitation and bleeding [137]. Due to the decreased blood loss and improved surgical field effects of TIVA, TIVA is generally preferred over inhalation anesthesia in studies of nose and sinus surgery [138]. Overall, there is unclear evidence as to whether TIVA or inhalation anesthesia is the best option for postoperative pain relief.

### 3.13. Non-Pharmaceutical Treatments

#### 3.13.1. Post-Procedure Ice

Post-procedure ice is often used as a method to reduce postoperative pain. Hilotherapy uses a chiller to receive continuous cooling and was found to decrease pain following rhinoplasty when used in comparison to standard ice packs [139]. Hilotherapy additionally prevents postoperative edema and ecchymosis after rhinoplasty [139,140]. The application of cooled silicone gel packs to the periorbital region significantly reduced edema, ecchymosis, and pain in the first three days after rhinoplasty [141]. Compared to ice in disposable latex gloves, cooling gel eye masks decreased facial pain and edema in rhinoplasty patients [142]. Notably, periorbital cooling did not improve pain relief in the first hour post procedure [141,142].

#### 3.13.2. Essential Oils

Essential oils are a consideration for reduction of pain. Lavender aromatherapy has been shown to reduce postoperative pain following cesarean section and inguinal hernia repair [143,144]. Additionally, in patients undergoing general otolaryngology surgery, lavender aromatherapy helps to reduce preoperative anxiety [145]. Patients with clinically significant burns were noted to have less pain after the inhalation of Damask rose essential oils compared to placebo [146]. The additional benefits of essential oils as an adjunct to pain management are their low cost and easy accessibility [146].

#### 3.13.3. Music

Music can decrease pain. A meta-analysis of 81 randomized controlled trials demonstrated that listening to music during general anesthesia significantly decreased pain and anxiety [147]. Listening to music 60 min/day in the post-septoplasty period led to a 27.2% and 64.9% reduction in nasal obstruction symptoms as measured by the NOSE score at the second and third follow-up visit, respectively [148].

#### 3.13.4. Hypnosis

Hypnosis was found to be effective in reducing pain. In a study on the effect of preoperative hypnosis in rhinoplasty, hypnosis reduced intraoperative remifentanil use and postoperative pain without affecting the surgical field [149]. However, there is limited access to hypnotists; hypnosis may not be available and accessible to most patients [149]. An additional consideration includes the patient’s hypnotic capacity [150].

## 4. Updated Protocol

**Figure 3:**
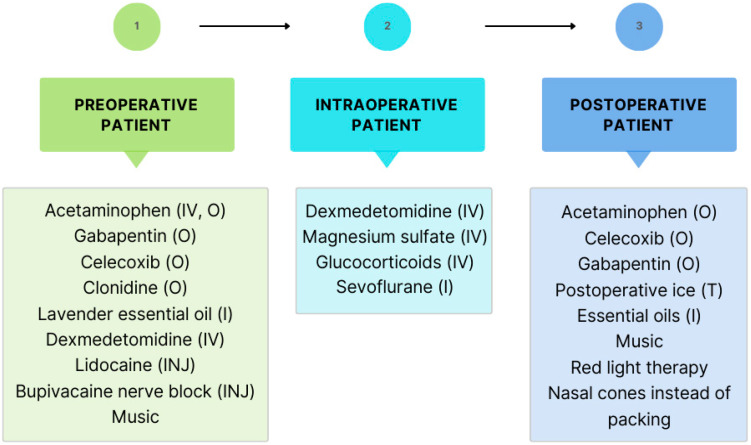
Updated protocol to reduce postoperative pain and opioid consumption. IV = intravenous, O = oral, INJ = injected locally, I = inhalation, T = topical.

As each patient will have different medical histories and personal preferences to consider, it is necessary to continue to update the multimodal analgesic protocol in rhinoplasty. The goal is to take a holistic approach to pain management by considering a patient’s background with reference to drug side effects and modes of administration, while also optimizing the magnitude of the effect on reducing opioid use and postoperative pain. The previously established protocol helped to determine which analgesic agent to use based on timing of administration: pre-dosing, intraoperatively, or postoperatively. Based on the updated details and evidence from the current literature review, this protocol has been further developed, including the following modes of administration: intravenous, oral, inhalation, and topical (Figure 3).

Options for managing pain in the preoperative phase includes intravenous or oral acetaminophen, oral gabapentin, oral celecoxib, oral clonidine, and lavender essential oil by inhalation, with intravenous dexmedetomidine, injected lidocaine, injected bupivacaine, and music in the updated protocol. In the intraoperative phase, non-opioid analgesia options include intravenous dexmedetomidine and glucocorticoids; intravenous magnesium sulfate and inhalation sevoflurane are updated additions to the protocol. For the postoperative patient, analgesic agents in the prior protocol included oral acetaminophen, oral celecoxib, oral gabapentin, nasal cones, essential oils, red light therapy, and postoperative ice; with music being the only major new addition to this phase of the protocol. Preferred analgesic agents include, as follows: preoperatively, 1000 mg of oral acetaminophen, 200 mg of oral celecoxib twice daily for 5 days, and 1200 mg of oral gabapentin; intraoperatively, 0.75 μg/kg of intravenous dexmedetomidine and 1–2 mg/kg injected lidocaine with an additional 2–4 mg/kg per hour or 1.5 cc total bupivacaine nerve block injected along the infraorbital area bilaterally and in the subnasal region; and postoperatively, 5 mg of oral acetaminophen and 400 mg of oral celecoxib. Potential alternatives for patients with special medical restrictions include substituting ibuprofen and avoiding celecoxib in patients with sulfa allergy, avoiding clonidine in patients with hypotension, and a preference for ketorolac in patients with liver metabolism dysfunction.

## 5. Conclusions

There is evidence that multimodal analgesia is an effective opioid-sparing technique in reducing pain following rhinoplasty. Well-established options with minimal side effects include acetaminophen, NSAIDs, and alpha-2 agonists. Notably, nerve blocks are shown to be a significantly effective analgesic agent for reducing postoperative pain in rhinoplasty; however, nerve block use in rhinoplasty needs to be further understood with additional studies. In general, surgeries of the nose and face are not as established with multimodal analgesia studies compared to abdominal and orthopedic surgery. Non-pharmaceutical additions to the analgesic regimen can include post-procedural ice, essential oils, music, and hypnosis, although these options can widely vary and are dependent on patient preference. When choosing specific analgesic agents, considerations include potential side effects, contraindications, and drug-specific mode of administration; thus, it is essential to obtain a thorough patient history, including diagnoses, medications, and allergies. Based on current evidence, minimization of opioid consumption after rhinoplasty can be safely achieved by preferentially prescribing a regimen of oral acetaminophen, oral celecoxib, and oral gabapentin in the preoperative phase, intravenous dexmedetomidine and intravenous lidocaine or bupivacaine during the intraoperative phase, and oral acetaminophen and oral celecoxib in the postoperative phase. Notably, side effects to be aware of are, as follows: acetaminophen—vomiting, abdominal pain, and hypotension; celecoxib—cardiovascular risk and gastrointestinal bleeding; gabapentin—delirium; dexmedetomidine—hypertension, bradycardia, and asystole. Lidocaine and bupivacaine are typically well-tolerated; however, allergic reactions are a consideration.

A limitation of this study is that much of the established knowledge on analgesics has resulted from studies in a variety of surgical fields, and not specifically rhinoplasty. These surgeries, such as cesarean section or gastrointestinal surgery, do not necessarily anticipate the same level of pain as rhinoplasty; therefore, the effects of analgesic agents on postoperative pain in these procedures might not be similarly observed in rhinoplasty. More studies are needed regarding the specific dosing and effects, in rhinoplasty, of the analgesic agents explored in this review; a future review will need to include the results of these studies for a more robust understanding of appropriate non-narcotic pain management regimens for rhinoplasty patients. Furthermore, many of the studies and analgesic agents reviewed in this article lacked observation of patient compliance rates, which is an important consideration when choosing the most optimal analgesic agent for a patient, as compliance will influence each drug’s overall perceived effectiveness.

New approaches to opioid-sparing analgesia are continuously being developed and updated with significant success in reducing patient pain. Future studies will not only need to consider the challenges and the specific pain experienced as a result of rhinoplasty but must also consider the risk of opioid misuse and analgesic agent effects at the individual level. It is necessary for plastic surgeons to continue to learn about these new techniques and implement them to properly manage each patient’s individual pain experience while limiting opioid consumption.
